# Practical Notes

**Published:** 1890-10

**Authors:** 


### PRACTICAL NOTES.

ASEPSIS IN VACCINATION.—Dr. Alfred Leach reports, in the *British Medical Journal*, a six years' experience in the antiseptic management of vaccination, of which the following are the principal precautions:

1. The lymph is collected on the day it is used.
2. The instruments, the patient's arm, and the lymph tube, before being opened, are washed in a disinfecting solution.
3. In four places small scarifications and valvular punctures are made with a Graefe's cataract knife that is charged with lymph.
4. When the wounds are perfectly dry they are dusted with bismuth, and a pad of dry lint is applied.
5. After the second day the parts are daily washed with a gentle current of cold water. No sponge is allowed to come in contact with the arm.

Results: He has had only one case of inflamed arm during the six years he has employed this plan of management.

